# Graphene Oxide Synthesis from Agro Waste

**DOI:** 10.3390/nano5020826

**Published:** 2015-05-20

**Authors:** Thirunavukkarasu Somanathan, Karthika Prasad, Kostya (Ken) Ostrikov, Arumugam Saravanan, Vemula Mohana Krishna

**Affiliations:** 1 Department of Nanoscience, School of Basic Sciences, Vels University, Pallavaram, Chennai, Tamil Nadu 600117, India; E-Mails: karthikaprasad@live.com (K.P.); saravanan.con@gmail.com (A.S.); krishnachem07@gmail.com (V.M.K.); 2 Center for Advanced Research and Development (CARD), Vels University, Pallavaram, Chennai, Tamil Nadu 600117, India; 3 Institute of Future Environments, Nanotechnology and Molecular Science, School of Chemistry, Physics and Mechanical Engineering, Queensland University of Technology, Brisbane, QLD 4000, Australia; E-Mail: kostya.ostrikov@qut.edu.au

**Keywords:** graphene oxide (GO), natural precursors, value-added reforming

## Abstract

A new method of graphene oxide (GO) synthesis via single-step reforming of sugarcane bagasse agricultural waste by oxidation under muffled atmosphere conditions is reported. The strong and sharp X-ray diffraction peak at 2θ = 11.6° corresponds to an interlayer distance of 0.788 nm (*d*_002_) for the AB stacked GOs. High-resolution transmission electron microscopy (HRTEM) and selected-area electron diffraction (SAED) confirm the formation of the GO layer structure and the hexagonal framework. This is a promising method for fast and effective synthesis of GO from sugarcane bagasse intended for a variety of energy and environmental applications.

## 1. Introduction

Graphene and related materials have attracted a great deal of recent interest owing to their unique structure and physical, chemical, thermal, and other properties [1]. Graphene’s two-dimensional (2D) hexagonal lattice consists of a network of *sp*^2^-bonded carbon atoms and represents the “thinnest material” which is stable in its free form [2]. Excellent electronic properties [3], thermal conductivity [4], and high surface area, combined with unusual mechanical properties and good dispersion performance, make graphene a promising candidate for structural modification of composite materials and several other applications [5,6,7,8].

Graphene oxide (GO) has also attracted major interdisciplinary attention because of the broad range of envisaged applications across several scientific and engineering fields including physics, chemistry, biology, and medicine [9,10,11,12,13]. The original method to synthesize GO is based on the addition of potassium chlorate to a slurry of graphite in fuming nitric acid [14]. This synthetic protocol can be improved by using concentrated sulfuric acid as well as fuming nitric acid and adding the chlorate in multiple aliquots over the course of the reaction [15]. In the commonly used Hummers’ method [16], graphite is oxidized throughout treatment of KMnO_4_ and NaNO_3_ in concentrated H_2_SO_4_ acid.

All these procedures involve the generation of NO_2_, N_2_O_4_, and ClO_2_, toxic gases which are also explosive. In the past few years, various techniques such as mechanical exfoliation techniques, chemical vapor deposition (CVD) techniques and other chemical techniques have been developed for the synthesis of graphene and graphene oxide materials. However, many of these techniques are highly sophisticated and expensive [17]. Most of the commercially available GO samples were synthesized by Hummers' method or a modified version of it. The cost of the commercially available graphene oxide per gram is approximately $200. In comparison with the Hummers' method, our method is more environment friendly as it avoids toxic gas emission during synthesis. The synthesis technique reported here is a quite efficient asset demonstrating a new procedure for the production of graphene oxide from agro waste in simple steps. This simple and low-cost process could lead to new opportunities for cost-effective production of GO [18,19].

Here we report on the synthesis of graphene oxide by directly oxidizing sugarcane bagasse under muffled atmosphere. The GO produced by this method is called SOMA-GO (sugarcane oxidized under muffled atmosphere for graphene oxide). Furthermore, the structural characteristics of the obtained product are confirmed by X-ray diffraction, Fourier transmission infrared spectroscopy, field emission scanning electron microscopy, high-resolution transmission electron microscopy, and Raman spectroscopy.

## 2. Experimental Section

### 2.1. Synthesis of GO from Agro Waste

**Scheme 1:**
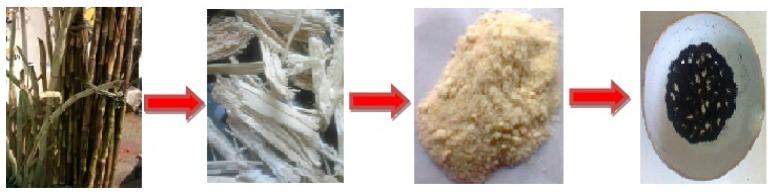
Schematic representation of graphene oxide synthesis from agro waste.

Sugarcane bagasse is a type of agricultural waste (agro waste). After juice was extracted, the remaining fiber was taken. The fiber was crushed and ground well in order to produce powder. This crushing and separating process was repeated several times to obtain fine powder. About 0.5 g of ground sugarcane bagasse powder mixed with 0.1 g of ferrocene was placed in a crucible and put directly into a muffle furnace at 300 °C for 10 min under atmospheric conditions. Next, the as-produced black solid was collected at room temperature. The black solid product was subjected to further analysis (Scheme 1).

### 2.2. Characterization of GO

The X-ray diffraction (XRD) pattern was obtained using a Bruker D8 Advance X-ray diffractometer (Berlin, Germany). This device is equipped with a Ni filter, and generates monochromated Cu-Kα radiation (λ = 0.154 nm) operated at 40 kV accelerating voltage and 30 mA current. The samples were scanned in step mode with a 2° min^−1^ scan rate. Fourier transform infrared spectroscopy (FT-IR), Avatar 370, Thermo Nicolet (Berlin, Germany) was carried out to study the bonding configuration in the synthesized graphene oxide. High resolution transmission electron microscopy (HRTEM) was performed using a JEOL JEM-3000F microscope (Tokyo, Japan) operating at 300 kV to study the microstructure of the GO structures. Field emission scanning electron microscopy was carried out using a JEOL JSM 7800F revealed the morphological features of the GO structure.

## 3. Results and Discussion

### 3.1. X-Ray Diffraction Measurements

**Figure 1:**
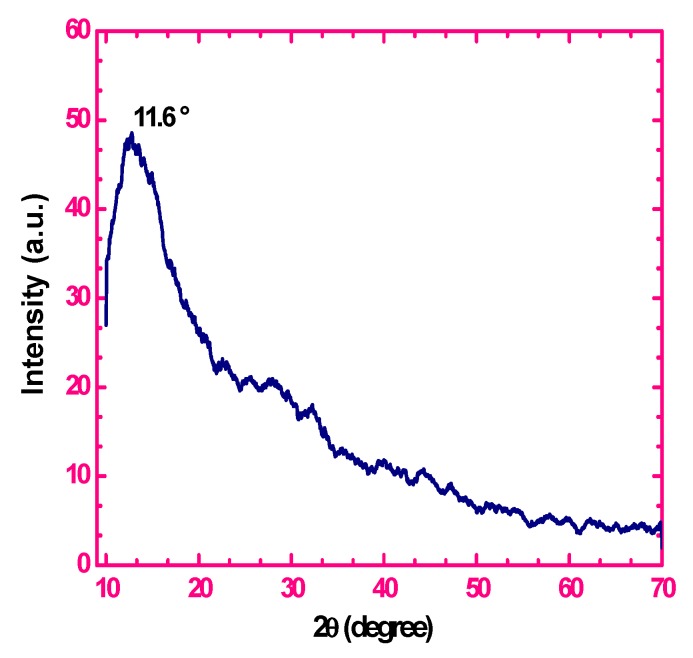
X-ray diffraction (XRD) pattern of graphene oxide.

The structural and chemical analysis of sugarcane bagasse is clearly described by previous investigators [20,21,22]. In this study, we have presented the XRD pattern of the GO which is shown in Figure 1. The peak at 2θ = 11.6° indicates that the agricultural sugarcane bagasse is fully oxidized into graphene oxide with the interlayer distance of 0.79 nm. The 2θ peaks of the GO obtained by the single-step oxidation of sugarcane bagasse are consistent with the results of previous studies [23,24]. This XRD pattern can be attributed to well graphitized two-dimensional structures made of GO sheets.

### 3.2. FT-IR Spectroscopy

**Figure 2:**
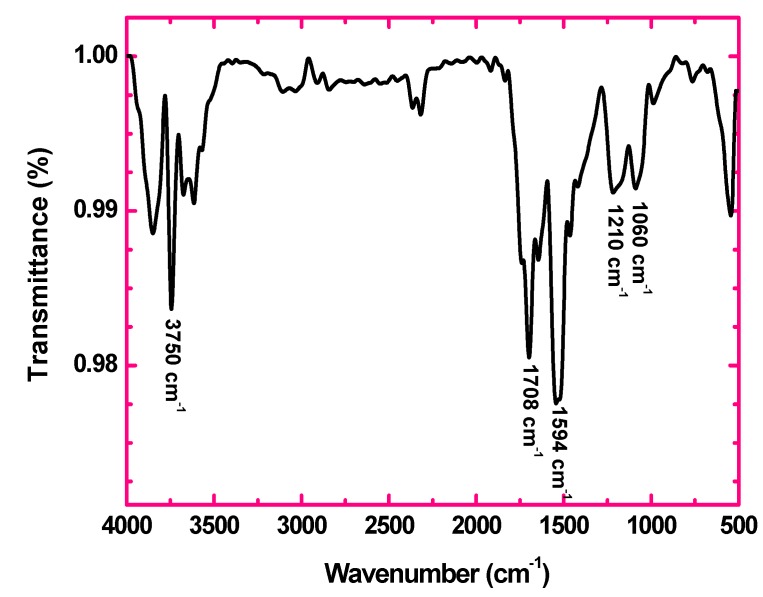
Fourier transform infrared spectroscopy (FT-IR) spectrum of graphene oxide.

The FT-IR spectrum of GO shown in Figure 2 confirms the introduction of oxygen containing groups such as functional hydroxyl, epoxy and carboxylic groups upon oxidation of sugarcane bagasse. The strong band at 1708 cm^−1^ is attributed to stretching vibration modes of C=O in carboxylic acid and carbonyl groups. The peak at 1594 cm^−1^ is assigned to the skeletal vibrations of un-oxidized graphitic domains. The band at 1060 cm^−1^ is assigned to C–O (epoxy) groups while the band at 1210 cm^−1^ is usually attributed to C–OH stretching vibrations [25,26,27]. The strong peak around 3500–4000 cm^−1^ can be attributed to the O–H stretching vibrations of the C–OH groups and water [27].

### 3.3. SEM and HRTEM Analysis

**Figure 3:**
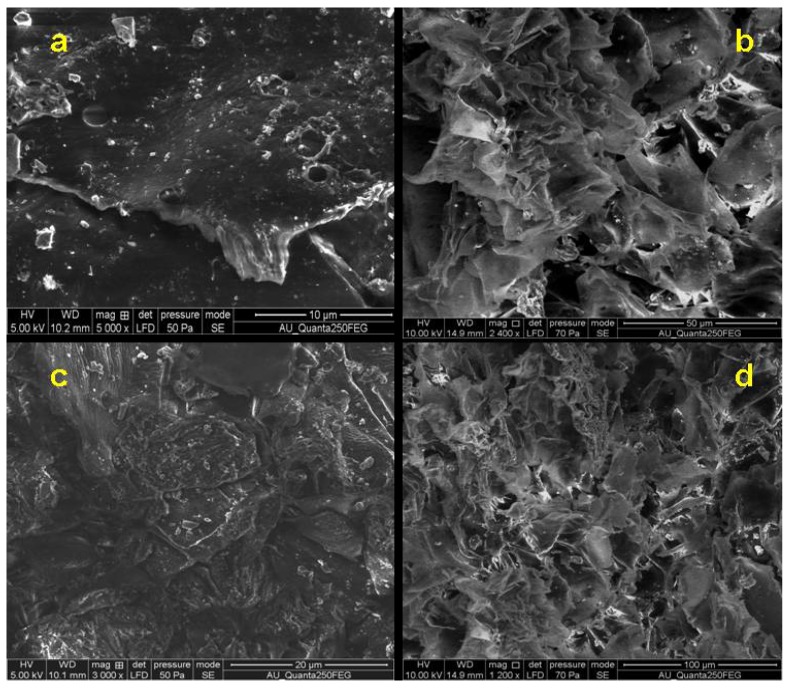
FESEM images of GO obtained from sugarcane bagasse.

The surface morphology of the graphene oxide (GO) produced by sugarcane bagasse oxidation is identified by FESEM. The SEM image of the GO shown in Figure 3a,c clearly reveals its sheet-like structure. The morphology of the synthesised GO resembles flake-like structures as shown in Figure 3b,d. Quite similar textures have been reported by other authors [28].

**Figure 4:**
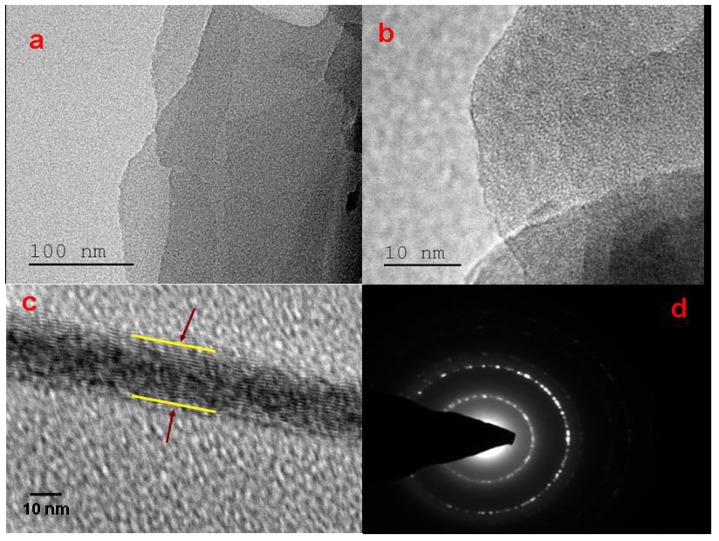
High-resolution transmission electron microscopy (HRTEM) images of GO obtained from sugarcane bagasse.

HRTEM micrographs of GO are shown in Figure 4. Bends wrinkles, and edges on graphene oxide nanosheets are seen in several places in Figure 4a,b. Various defects and functional groups containing *sp*^3^ hybridized carbon atoms may also be introduced during the oxidation process. Figure 4c shows that the flakes contain a few layers of graphene oxide. The selected area of electron diffraction (SAED) pattern shown in Figure 4d indicates the presence of a polycrystalline structure corresponding to graphene oxide [29,30,31,32,33]. Thus, transmission electron microscopy (TEM) analysis confirms the presence of graphene oxide in the oxidized carbon samples.

### 3.4. Raman Spectroscopy

**Figure 5:**
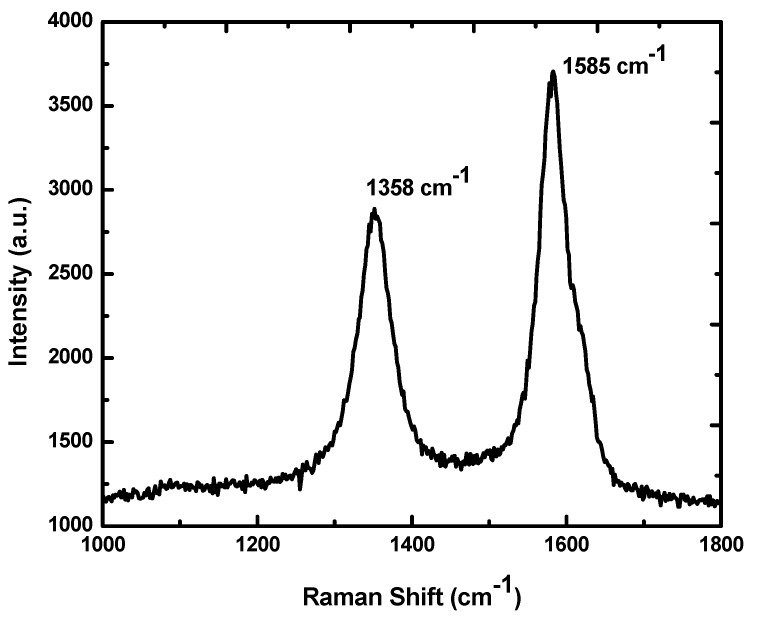
Raman spectrum of GO obtained from sugarcane bagasse.

The Raman spectrum of graphene oxide synthesized from sugarcane bagasse is shown in Figure 5. The spectrum of GO displays two prominent peaks at 1358 and 1585 cm^−1^, which are attributed to the local defects (found at the edges of graphene sheets) and the *sp^2^* graphitized structure, respectively. The *G* peak formation is due to the double degenerated zone center *E*_2_*g* mode [34,35]. The functionality and reduction in particle size of *sp*^2^ plane domains are possibly due to the extensive oxidation of sugarcane bagasse [24]. The two *D* and *G* bands at 1358 cm^−1^ and 1585 cm^−1^ [36] indicate the clear *sp*^2^ carbon hybridization in the observed multi-layer stacks [31]. Thus, *I_D_*/*I_G_* peak intensity ratios are assigned to lower defects/disorders. Raman spectra show an intensity ratio of *I_D_*/*I_G_* at 0.76 for GO which is in line with previous investigations [24,37,38].

## 4. Conclusions

A simple and rapid method is shown here to effectively convert solid sugarcane bagasse waste into value-added graphene oxide. The produced graphene oxide presents a well-graphitized structure. In comparison with the commonly used methods, our method is more environment friendly as it avoids toxic gas emission during the synthesis. This simple and low-cost synthesis process could lead to new opportunities for cost-effective production of graphene-based materials for gas sensors, energy storage, and other functional devices.

## References

[1] Chae H.K., Siberio-Perez D.Y., Kim J., Go Y., Eddaoudi M., Matzger A.J., O’Keeffe M., Yaghi O.M. (2004). A route to high surface area, porosity and inclusion of large molecules in crystals. Nature.

[2] Novoselov K., Geim A., Morozov S., Jiang D., Zhang Y., Dubonos S., Grigorieva I., Firsov A. (2004). Electric field effect in atomically thin carbon films. Science.

[3] Schwierz F. (2010). Graphene transistors. Nat. Nanotechnol..

[4] Balandin A.A., Ghosh S., Bao W., Calizo I., Teweldebrhan D., Miao F., Lau C.N. (2008). Superior thermal conductivity of single-layer graphene. Nano Lett..

[5] Song H., Zhang L., He C., Qu Y., Tian Y.F., Lv Y. (2011). Graphene sheets decorated with SnO_2_ nanoparticles: *In situ* synthesis and highly efficient materials for cataluminescence gas sensors. J. Mater. Chem..

[6] Si Y.C., Samulski E.T. (2008). Exfoliated graphene separated by platinum nanoparticles. Chem. Mater..

[7] Lu G., Park S., Yu K., Ruoff R.S., Ocola L.E., Rosenmann D., Chen J. (2011). Toward practical gas sensing with highly reduced graphene oxide: A new signal processing method to circumvent run-to-run and device-to-device variations. ACS Nano.

[8] Lee C., Wei X., Kysar J.W., Hone J. (2008). Measurement of the elastic properties and intrinsic strength of monolayer grapheme. Science.

[9] Georgakilas V., Otyepka M., Bourlinos A.B., Chandra V., Kim N., Kemp K.C., Hobza P., Zboril R., Kim K.S. (2012). Functionalization of graphene: Covalent and non-covalent approaches, derivatives and applications. Chem. Rev..

[10] Novoselov K.S., Fal’ko V.I., Colombo L., Gellert P.R., Schwab M.G., Kim K. (2012). A roadmap for grapheme. Nature.

[11] Chung C., Kim Y.K., Shin D., Ryoo S.R., Hong B.H., Min D.H. (2013). Biomedical applications of graphene and graphene oxide. Acc. Chem. Res..

[12] Mao H.Y., Laurent S., Chen W., Akhavan O., Imani M., Ashkarran A.A., Mahmoudi M. (2013). Graphene: Promises, facts, opportunities, and challenges in nanomedicine. Chem. Rev..

[13] Eigler S., Hu Y., Ishii Y., Hirsch A. (2013). Controlled functionalization of graphene oxide with sodium azide. Nanoscale.

[14] Brodie B.C. (1859). On the atomic weight of graphite. Philos. Trans. R. Soc. Lond..

[15] Staudenmaier L. (1898). Verfahren zur Darstellung der Graphitsäure. Ber. Dtsch. Chem. Ges..

[16] Hummers W.S., Offeman R.E. (1958). Preparation of graphitic oxide. J. Am. Chem. Soc..

[17] Guo S., Dong S. (2011). Graphene nanosheet: Synthesis, molecular engineering, thin film, hybrids, and energy and analytical applications. Chem. Soc. Rev..

[18] Seo D.H., Rider A.E., Kumar S., Randeniya L.K., Ostrikov K. (2013). Vertical graphene gas- and bio-sensors via catalyst-free, reactive plasma reforming of natural honey. Carbon.

[19] Seo D.H., Han Z.J., Kumar S., Ostrikov K. (2013). Structure-controlled, vertical grapheme-based, binder-free electrodes from plasma-reformed butter enhance super capacitor performance. Adv. Energy Mater..

[20] Chandel A.K., Antunes F.A.F., Anjos V., Bell M.J.V., Rodrigues L.N., Polikarpov I., de Azevedo E.R., Bernardinelli O.D., Rosa C.A., Pagnocca F.C. (2014). Multi-scale structural and chemical analysis of sugarcane bagasse in the process of sequential acid-base pretreatment and ethanol production by Scheffersomycesshehatae and Saccharomyces cerevisiae. Biotechnol. Biofuels.

[21] Rezende C.A., de Lima M.A., Maziero P., de Azevedo E.R., Garcia W., Polikarpov I. (2011). Chemical and morphological characterization of sugarcane bagasse submitted to a delignification process for enhanced enzymatic digestibility. Biotechnol. Biofuels.

[22] Velmurugan R., Muthukumar K. (2011). Utilization of sugarcane bagasse for bioethanol production: Sono-assisted acid hydrolysis approach. Bioresour. Technol..

[23] Muralidharan M.N., Ansari S. (2013). Thermally reduced graphene oxide/thermoplastic polyurethane nanocomposites as photomechanical actuators. Adv. Mater. Lett..

[24] Kellici S., Acord J., Ball J., Reehal H.S., Morgan D., Saha B. (2014). A single rapid route for the synthesis of reduced graphene oxide with antibacterial activities. RSC Adv..

[25] Marcano D.C., Kosynkin D.V., Berlin J.M., Sinitskii A., Sun Z., Slesarev A., Alemany L.B., Lu W., Tour J.M. (2010). Improved synthesis of graphene oxide. ACS Nano.

[26] Xu Y., Bai H., Lu G., Li C., Shi G. (2008). Flexible graphene films via the filtration of water-soluble noncovalent functionalized graphene sheets. J. Am. Chem. Soc..

[27] Verma S., Mungse H.P., Kumar N., Choudhary S., Jain S.L., Sain B., Khatri O.P. (2011). Graphene oxide: An efficient and reusable carbocatalyst for aza-Michael addition of amines to activated alkenes. Chem. Commun..

[28] Satish B., Venkata Rao K., Shilpa Chakra C.H., Tejaswi T. (2013). Synthesis and characterization of graphene oxide and its antimicrobial activity against klebseilla and staphylococus. Int. J. Adv. Biotechnol. Res..

[29] Sun Z., Yan Z., Yao J., Beitler E., Zhu Y., Tour J.M. (2010). Growth of graphene from solid carbon sources. Nature.

[30] Roy M., Kusurkar T.S., Maurya S.K., Meena S.K., Singh S.K., Sethy N., Bhargava K., Sharma R.K., Goswami D., Sarkar S. (2014). Graphene oxide from silk cocoon: A novel magnetic fluorophore for multi-photon imaging. 3 Biotech.

[31] Bo Z., Shuai X., Mao S., Yang H., Qian J., Chen J., Yan J., Cen K. (2014). Green preparation of reduced graphene oxide for sensing and energy storage applications. Sci. Rep..

[32] Mao S., Pu H., Chen J. (2012). Graphene oxide and its reduction: Modeling and experimental progress. RSC Adv..

[33] Mao S., Yu K., Cui S., Bo Z., Lu G., Chen J. (2011). A new reducing agent to prepare single-layer, high-quality reduced graphene oxide for device applications. Nanoscale.

[34] Vidano R.P., Fischbach D.B., Willis L.J., Loehr T.M. (1981). Observation of Raman band shifting with excitation wavelength for carbons and graphites. Solid State Commun..

[35] Tuinstra F., Koenig J.L. (1970). Raman spectrum of graphite. J. Chem. Phys..

[36] Chun O.W., Chen M.L., Zhang K., Zhang F.J. (2010). The effect of thermal and ultrasonic treatment on the formation of graphene-oxide nanosheets. J. Korean Phys. Soc..

[37] Kaniyoor A., Ramaprabhu S. (2012). A Raman spectroscopic investigation of graphite oxide derived graphene. AIP Adv..

[38] Eigler S., Dotzer C., Hirsch A. (2012). Visualization of defect densities in reduced graphene oxide. Carbon.

